# Biological Efficacy of Essential Oils and Plant Extracts of Cultivated and Wild Ecotypes of *Origanum vulgare* L.

**DOI:** 10.1155/2020/8751718

**Published:** 2020-04-06

**Authors:** Sumira Jan, Megna Rashid, Elsayed F. Abd_Allah, Parvaiz Ahmad

**Affiliations:** ^1^Department of Basic Science & Humanities, SKUAST-K Shalimar, Srinagar, Jammu and Kashmir 190006, India; ^2^Faculty of Forestry, SKUAST-K, Shalimar, Srinagar, Jammu and Kashmir 190006, India; ^3^Department of Plant Production, Faculty of Food & Agricultural Sciences, King Saud University, Riyadh, Saudi Arabia; ^4^Botany and Microbiology Department, College of Science, King Saud University, P.O. Box. 2460, Riyadh 11451, Saudi Arabia; ^5^Department of Botany, S. P. College, Srinagar 190001, Jammu and Kashmir, India

## Abstract

Current study describes discrepancy in biological efficacy of methanolic and ethanolic extracts and essential oil procured from cultivated and wild accessions of *Origanum vulgare.* Simultaneously, quantification of carvacrol, thymol, caryophyllene, ocimene, and terpinen-4-ol contents was determined via GC-MS and GC in both accessions. The results revealed significantly a higher antioxidant potential by methanolic extracts displaying IC_50_ of 19.9 *μ*g/ml compared to essential oil with IC_50_ of 10 *μ*g/ml, and ethanolic extracts were found to be less effective even at the concentration of 3 *μ*g/ml. However, essential oil from wild and cultivated accessions of *O. vulgare* exhibited significantly high antimicrobial activity against all 39 bacteria, 16 fungi, and 2 yeast species tested due to higher concentrations of carvacrol and thymol as revealed by GC analysis. Inhibition of tyrosinase activity in a C6 cell line displayed 81.0%–87.0% depigmentation potential of the methanolic extracts, while ethanolic extracts revealed a maximum of 88.54–99.02% inhibition of reactive oxygen species (ROS) in H_2_O_2_-treated cells. Hence, the study determines efficacy of essential oil against microbial pathogenesis, methanolic extracts as potent depigmentation agents, and ethanolic extracts as potent free radical scavenger.

## 1. Introduction

*Origanum* (*Lamiaceace*), known by the common name oregano, is an herbaceous perennial indigenous to Europe, North Africa, and temperate regions of Asia [1, 2]. *Origanum* species grow copiously on mountainous areas and hilly areas with extensive ranges of altitudes [3]. The immense variability in volatile as well as nonvolatile fractions of the genus *Origanum* that can thrive in varied climatic belts gives them a strong utility in agriculture, medicine, and cosmetics, as a flavoring and aromatic agent [4–6]. In addition to relevance in the medicine and agriculture, essential oil from *O. vulgare* can be used as food disinfectant. Food infestation caused by microbes is a major predicament in the world, including in well-developed countries such as the USA [7, 8]. Numerous bacteria (*Escherichia coli*, *Enterobacter* spp., *Bacillus* spp., *Salmonella* spp., *Staphylococcus aureus*, *Klebsiella pneumoniae*, *Listeria monocytogenes*, and *Campylobacter jejuni*), yeast (*Candida* spp. and *Zygosaccharomyces* spp.), and fungi (*Fusarium* spp., *Aspergillus* spp., *Rhizopus* spp., and *Penicillium* spp.) are common food pathogens that cause food spoilage [9]. Oregano has been assessed for its antioxidant and antimicrobial characteristics with particular relevance to food preservation [10, 11]. The popularized naturally preserved meat is samarella containing major fraction of oregano [12]. Moreover, oregano essential oil has been used as a major food additive in European Union for health benefits [13, 14].

Chemical preservatives have used to avoid food spoilage [15, 16]. However, since chemical preservation leads to constraints in consumer acceptability and other health-related issues, researchers have begun to investigate the use of organic and natural preservatives [17, 18].

Oregano is a flowering plant in the mint family (Lamiaceace). It is native to temperate Western and Southwestern Eurasia and Mediterranean region. Oregano is a perennial herb, growing from 20–80 cm in height with alternate leaves 1–4 cm. The flowers are purple, long, produced in erect spikes [10]. Indeed, the tribal people of western Himalayan belt use *O. vulgare* against flatulence, diaphoresis, and cough, to promote menstrual discharge, energize the body, increase appetite, and as a tonic [11, 19–21]. This herb possesses enough potential, if utilized to its maximum potential through specific breeding and laboratory techniques, to meet the present nutritional needs and secure the future demands [22].

Due to the introduction of exotic species of oregano into the Himalayan western belt, there exists a huge amount of phytochemical variation and genetic polymorphisms between *O. vulgare* plants and no precise data are present [20]. The goal of the current research was to determine the comparative biological efficacy of *O. vulgare* essential oil and extracts procured from diverse wild and cultivated accessions thriving in the mountains fields of western Himalayan belt. Although there are 42 species of genus *Origanum*, its ecotype from Kashmir has not yet been subjected to phytochemical characterization, and its biological efficacy has not been studied. Moreover, the imprecise taxonomy and assessment of genus *Origanum* extracts and its essential oils utilized as dietary supplements or for medicinal purposes in developed countries have made it necessary to screen our native species to verify its validity and product quality so that it can be used as a valuable nutraceutical.

Keeping in mind the significance of the herb, the present research was conducted (1) to determine discrepancies in essential oil components in diverse ecotypes of *O. vulgare* using GC and GC-MS and (2) to evaluate variation and validate the divergence in biological efficacy of plant extracts and essential oils procured from diverse ecotypes of *O. vulgare* L.

## 2. Materials and Methods

### 2.1. Survey and Collection of Material

The aerial parts of *O. vulgare* were procured from seven wild and three cultivated populations of *O. vulgare* L. collected from diverse microclimatic zones in the Kashmir Himalayas, as shown in Table 1.

Collected samples were verified by Prof. Gurcharan Singh, University of Delhi. 1,1-Diphenyl-2-picrylhydrazyl (DPPH), 2,2′-azinobis (3-ethylbenzothiazoline-6-sulfonic acid) diammonium salt (ABTS^+^), 2,5,7,8-tetramethylchroman carboxylic acid (trolox), 2-thiobarbituric acid (TBA), 4-hydroxyphenyl b-D-glucopyranoside (arbutin), ethylene diamine-tetraacetic acid disodium salt dihydrate (EDTA), hydrogen peroxide (H_2_O_2_), L-ascorbic acid, trichloracetic acid (TCA), and 3-(2-pyridyl)-5,6-diphenyl-1,2,4-triazine-4′,4^″^-disulfonic acid monosodium salt (ferrozine) were purchased from Sigma Aldrich (St. Louis, MO). All reference standards were of GC grade, and other chemicals were of analytical grade.

### 2.2. Preparation of the Extracts

#### 2.2.1. Isolation of the Essential Oil

Shoots harvested from diverse ecotypes of *O vulgare* L. were shade-dried and subjected to water distillation using a Clevenger-type apparatus (yield 2.31% v/w). The essential oil obtained was dried with anhydrous sodium sulfate and then stored at 4°C until used in GC and GC-MS analysis.

#### 2.2.2. Preparation of the Methanolic and Ethanolic Extracts

Leaves (300 g) harvested from different ecotypes of *O. vulgare* were first shade-dried and then milled to fine powder. From this powder, 3 g was dispensed in 80 ml petroleum ether using a Soxhlet extractor to remove lipid and protein moieties. After recovery of the solvent, the extract was dissolved in 20 ml HPLC grade methanol or ethanol. The resulting extracts were decanted using Whatman filter paper (No. 1) and concentrated in a rotary evaporator. The remaining residue was kept at 0°C until further investigation.

#### 2.2.3. GC-MS and GC Analysis Conditions

The essential oil was subjected to GC-MS (Hewlett Packard 5890 II GC, fitted with a HP-5 MS capillary column and a HP 5972 mass selective detector). An electron ionization system with 70 eV ionization energy was employed; helium was the carrier gas, and a column flow rate of 1.21 ml/min was used. The column temperature was set to 80°C for 3 min, then steadily increased to 200°C, and finally raised to 280°/min rate for 30 min. Samples were diluted at 1 : 100 in methanol, and 1.0 *μ*l was injected manually in the split-less mode. The resulting spectra were compared with already identified components by using the information in the NIST library and previous literature [23]. These results were compared with reference standards of carvacrol, terpene 4-ol, *β*-caryophyllene, ocimene, sabinene, and thymol in order to determine variation among the major compounds of the essential oil. The variations were established by comparing relative retention indices on nonpolar phases with reference compounds, as was presented in a previous study [23].

### 2.3. Antioxidant Activity

#### 2.3.1. DPPH Assay

The antioxidant potential of the extracts was determined via an assay based on the stable radical DPPH, according to the procedure described by Burits and Bucar [24]. In brief, 20 *μ*l of various concentrations of methanolic and ethanolic extracts and 10 *μ*l of essential oil was mixed with 0.006% DPPH solution. Following 30 min incubation at 25°C, absorbance was recorded at 517 nm using a blank as a reference. The percentage inhibition of DPPH (*I*%) was calculated as follows:

(1) $$\begin{array}{c} I\%=\left(\frac{A_{\text{blank}}-A_{\text{sample}}}{A_{\text{blank}}}\right)\;\times 100\left(I\right), \end{array}$$

where *A*_blank_ is the absorbance of the control reaction (containing all reagents except the test compound) and *A*_sample_ is the absorbance of the test compound. A graph was plotted with extract concentration versus inhibition percentage to yield concentration that caused a 50% inhibition (IC50). Butylated hyroxytoluene (BHT) was used as a standard compound, and each experiment was repeated three times. The results were then expressed as equivalent antioxidant capacity of standards, i.e., mg standard equivalents/mg dry wt. of extract:

(2) $$\begin{array}{c} \frac{\text{IC}50\;\mathrm{standard}\;\left(\text{mg}/\text{mL}\right)}{\text{IC}50\;\mathrm{sample}\;\left(\text{mg}/\text{mL}\right)}=\frac{X\text{ mg standard equivalent}}{\text{mg dry weight }\left(\text{II}\right)}. \end{array}$$

#### 2.3.2. *β*-Carotene-Linoleic Acid Assay

This assay was used to estimate the antioxidant potential of the plant extracts by observing the change in color of *β*-carotene due to linoleic acid after oxidation [25]. In this assay, an emulsion was prepared using 0.5 mg *β*-carotene dispensed in 1 ml chloroform (HPLC grade), to which 25 *μ*l linoleic acid and 200 mg Tween 40 were added. The resultant solution was concentrated using vaporizing chloroform in a vacuum evaporator, after which 100 ml oxygenated distilled water was added and mixed vigorously. The resulting emulsion was dispensed in a test tube filled to the 1500 *μ*l mark, and 150 *μ*l of both methanolic and ethanolic extracts was added. The emulsion was then kept in an incubator at 25°C for 48 h, and then the absorbance was recorded at 490 nm. To prepare the positive control BHT and the ethanol blank, the exact same procedure was repeated.

#### 2.3.3. ABTS^+^ Radical Cation-Scavenging Activity

The radical-scavenging efficacy of the essential oil and methanolic and ethanolic extracts was estimated according to the procedure described by Re et al. [26]. In brief, ABTS^+^ reagent was dispersed in water to make a 10 mM stock solution, which was mixed with 5 mM potassium persulfate to form the ABTS^+^ radical cation. The resulting solution was kept in the dark for 12–16 h prior to use. To equilibrate the spectrophotometer, ABTS^+^ radical cation solution was diluted with ethanol to validate absorbance at 734 which was recorded as 0.89 ± 0.01 at 30°C. After calibration, 15 *μ*l of the methanolic and ethanolic extracts and 10 *μ*l of the essential oil were mixed with 3 ml of ABTS^+^ radical solution. The mixture was kept for 30 min, and then, absorbance was recorded at 734 nm. For the trolox assay, L-ascorbic acid was used as the reference. Extracts exhibiting a similar percentage variation in ABTS^+^ absorbance as measured for 0.7 mM trolox were taken as one trolox equivalent antioxidant capacity (TEAC).

#### 2.3.4. FRAP Assay for Reducing Power

The reducing power of the essential oil and the methanolic and ethanolic extracts of *O. vulgare* was estimated according to the method described by Barreira et al. [27]. In brief, 10 *μ*l essential oil and 25 *μ*l methanolic and ethanolic extracts of *O. vulgare* L. from each accession were dispersed in a mixture containing sodium phosphate buffer (0.3 ml, 0.5 mM, pH 6.8) and potassium ferricyanide (4.5 ml, 1%). Following incubation at 50°C for 30 min, 1 ml of 15% TCA (w/v) was added. Then, the mixture was subjected to centrifugation at 8,000 ×g for 20 min. After centrifugation, 5 ml of supernatant was dissolved with 8 ml deionized water and 12 ml (0.4%) of ferric chloride, and absorbance was recorded at 700 nm. Elevated absorbance levels signified a higher reducing power. The final result was recorded using L-ascorbic acid as the standard expressed as L-ascorbic acid equivalents per g of sample.

#### 2.3.5. Metal Chelating Activity

The metal chelating activities of the methanolic and ethanolic extracts of *O. vulgare* L. were obtained using a method modified of that described by Decker and Welch [28]. Approximately 15 *μ*l methanolic and ethanolic extracts and 35 *μ*l of FeCl_2_ (4 mM) were kept still for 10 min, and after adding, 50 *μ*l of 5 mM ferrozine and deionized water were mixed to make a final volume up to 1 ml. After vigorous mixing and incubation at 25°C for 20 min, absorbance was recorded at 562 nm. EDTA was used as a reference standard, and the capacity to chelate ferrous ions was determined using the following formula:

(3) $$\begin{array}{c} \text{chelating activity }\left(\%\right)=\left(\frac{1-A_{\text{sample}}}{A_{\text{control}}}\right)\times 100. \end{array}$$

#### 2.3.6. Cell Culture

The studies were performed on a C6, *Rattus norvegicus* glioma-derived cell line purchased and maintained in cell culture as recommended by ATCC (LGC Promochem GmbH, Wesel, Germany). The cells were cultured in Dulbecco-modified Eagle's medium supplemented with 10% fetal bovine syndrome (FBS) and 100 units penicillin-streptomycin and preserved at 37°C in a moistened incubator with 5% CO_2_ atmosphere. All the experiments reported here were performed under the following protocol: cells were grown to 85–95% confluency, trypsinized, and subcultured in culture dishes at a concentration of 2 × 10^4^ cells/ml. The cells were continually maintained in a humidified incubator with 5% CO_2_ at 37°C.

#### 2.3.7. Measurement of ROS Production

Intracellular production of ROS was determined using a color transition of 2,7-dichlorofluorescein diacetate (DCFH-DA) to dichlorodihydrofluoroscein (DCF) after treatment with H_2_O_2._ Methanolic and ethanolic extracts (15 *μ*l each) were dispersed over a C6 cell line derived from *Rattus norvegicus* glioma cell line subcultured at a concentration of 2 × 10^4^ cells/ml and incubated with the reference compounds *L*-ascorbic acid and trolox for 24 h. Cells were separated from trypsin-EDTA and thoroughly washed with PBS after incubation. Both plant extracts and control cells were then dispersed in 10 ml PBS containing DCFH-DA for 40 min at 37°C and finally incubated with 0.3 mM H_2_O_2_ for 40 min at 37°C. To estimate the ROS scavenging activity, fluorescence intensities of DCFH were measured using an excitation wavelength (*E*_x_) of 504 nm and emission wavelength (*E*_m_) of 524 nm.

#### 2.3.8. Antimicrobial Activity

Essential oil and methanolic extracts of the different cultivated and wild accessions of *O. vulgare* were analyzed against a collection of 53 microbes, including 30 bacteria and 21 fungi and yeast species (Tables 2–5), provided by the Department of Microbiology, Faculty of Food sciences, University of Kashmir. Identification of microbes was confirmed using the Microbial Identification System in Biochemistry at the Sher-i-Kashmir Institute of medical Sciences.

#### 2.3.9. Disc-Diffusion Assay

Essential oil and methanolic extracts were filtered through 0.45 *μ*m Millipore filters and then analyzed for antimicrobial activity using a disc diffusion assay according to a procedure described previously [29]. In brief, 150 *μ*l suspension containing 123 CFU/ml bacteria, 116 CFU/ml yeast, and 116 spore/ml fungi was inoculated on nutrient agar (NA), sabouraud dextrose agar (SDA), and potato dextrose agar (PDA) medium, respectively. Once microbial growth had reached a disc size of 6 mm diameter, 10 *μ*l essential oil or the 25 mg/ml extracts (300 *μ*g/disc) were added to the inoculated agar. Methanol was used as a solvent for the preparation of positive and negative controls, as described in the footnotes of Tables 4–6. Incubation of inoculated plates was carried out at 37°C for 24 h for bacteria, 48 h for yeast, and 72 h for fungi isolates. Plant-associated microorganisms were incubated at 27°C. Antimicrobial activity was analyzed via measurement of the zone of inhibition against the test microbes. Each assay in this experiment was repeated twice.

#### 2.3.10. Microwell Dilution Assay

The susceptibilities of microbial strains to essential oil and methanolic extracts were estimated through determination of minimal inhibition concentration (MIC) values. Broth culture kept for 12 h produced inocula of bacterial strains in suspension, which was regulated to 0.5 McFarland standard turbidity. Methanolic extracts (500 *μ*g/ml) were prepared by dissolving the pure extract in 10% dimethylsulfoxide (DMSO). This solution was then diluted again to generate concentrations ranging from 7.8 to 500 *μ*g/ml in 10 ml sterile nutrient containing test tubes. MIC values of methanolic extract and essential oil from diverse ecotypes of *O. vulgare* L. against bacterial strains and yeast isolates were evaluated using a microwell dilution following a method described previously [30], with minor modifications. In brief, a 96-well plate was seeded with 95 *μ*l nutrient broth and 5 *μ*l of inoculum in each well. Essential oil and methanolic extracts (100 *μ*l; 500 *μ*g/ml) were then added to the first wells. The last well, containing 195 *μ*l nutrient broth and 5 *μ*l of the inoculum on each strip, was employed as a negative control. Cefepime hydrochloride (500–7.8 *μ*g/ml) was prepared in nutrient broth and employed as standard drug for positive control. The plates were enclosed with a sterilized plate sealer. Plates containing content were vigorously centrifuged at 18 gyre for 15 s and then incubated for 24 h at room temperature. Growth of microbes in each media was determined via measurement of absorbance (Abs) at 600 nm using a microplate reader using 5 *μ*l samples with nutrient agar medium in clear wells as a negative control. The methanolic extract was screened two times against each microbe.

#### 2.3.11. MIC Agar Dilution Assay

Essential oil and methanolic extracts of *O. vulgare* L. were analyzed for MIC values of fungal isolates using the agar dilution assay according to the method described by Gul et al. [31]. PDA medium containing tween 20 (0.5%, v/v; Sigma) was supplemented with essential oil and methanolic extracts at a suitable volume to produce concentrations of 7.8–500 *μ*g/ml. Resulting PDA agar solutions were then dispensed in Petri plates followed by continuous vortexing. The plates were spot inoculated with 5 *μ*l (116 spore/ml) of each fungal isolate. Amphotericin B was employed as a reference antifungal drug. The inoculated plates were incubated at 27 and 37°C for 72 h for plant and pathogenic fungi isolates, respectively. After the incubation period, the plates were assessed for the presence or absence of growth. MIC values were evaluated as the lowest concentration of the essential oil where absence of growth was documented. Each test was repeated at least twice.

#### 2.3.12. Statistical Analysis

All analyses were done at least in triplicate, and these values were then presented as average values along with their standard derivations. Data were analyzed using the SAS software. Statistical comparisons were performed with principal coordinate analysis for the disparity among different antioxidant assays.

## 3. Results

### 3.1. Chemical Composition of the Essential Oil

Essential oil analysis was carried out via GC-MS and comparison of the results with a data library. As observed in Table 7, GC/MS analysis of the essential oil led to the identification of 39 compounds representing a minimum of 52.99% oil content in cultivated accessions to 91.18% content in wild accessions of *O. vulgare* L. Predominant constituents in the volatile fraction of essential oil of *O. vulgare* L. in both cultivated and wild accessions included carvacrol (52.99–91.18%), *β*-caryophyllene (0.04–1.87%), terpinen-4-ol (0.02–0.32%), limonene (0.03–0.19%), thymoquinone (0.02–0.19%), and (Z)-*β* Ocimene (0.12–0.18%). *trans*-Sabinene hydrate exhibited the maximum variation with wild accession species possessing the highest concentrations (0.9–1.43%) and cultivated accessions possessing the lowest concentrations (0.01–0.04%), as shown in Table 7.

#### 3.1.1. Free Radical-Scavenging Capacity and Reducing Power of Wild and Cultivated Accessions of *O. vulgare* L

As shown in Table 8, using the data from the DPPH assay, essential oil from wild accessions had IC_50_ of 2021–4100 *μ*g/ml, while essential oil from cultivated accessions had IC_50_ of 334–1300 *μ*g/ml. Methanolic extracts of wild accessions had the highest IC_50_ (7.8–17.9 *μ*g/ml), while cultivated accessions had lower values (0.9–4.9 *μ*g/ml). Ethanolic extracts of wild accessions had IC_50_ values of 51.7–71.1 *μ*g/ml, while ethanolic extracts from cultivated accessions had IC_50_ values of 24.1–44.5 *μ*g/ml. Percentage DPPH scavenging activities were in accordance with the calculated IC_50_ values in all samples (Table 8).

Table 8 demonstrates a concentration-dependent pattern in both the oil and extracts. ABTS^+^ radical-scavenging potential of essential oil, methanolic/ethanolic extracts, and standard L-ascorbic acid and trolox were remarkable, displaying capacities to change the absorbance of the ABTS^+^ comparable to 1 mM Trolox with maximum values of 192.4–384.40%, 37.25–80.39%, and 55.87–120.59% for samples from wild accessions of *O. vulgare*, respectively. Values for essential oil and methanolic and ethanolic extracts from cultivated accessions were 15–49%, 8–25%, and 12–37%, respectively. In both cases, the essential oil exhibited a higher radical-scavenging capacity than the ethanolic and methanolic extracts (Table 9).

**Figure 1:**
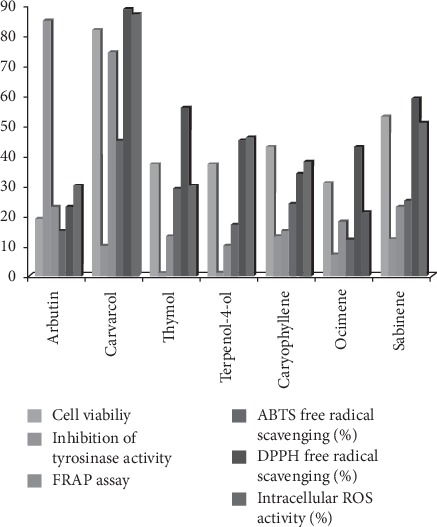
Inhibition of cellular tyrosinase, cell viability, ABTS, DPPH, and FRAP free radical-scavenging, and intracellular ROS activities (%). Treatments with 10 *μ*g/ml of each compound on C6 cell line glioma-derived cell line for 72 h; cell viability was determined by the MTT assay. Each value is presented as mean ± SD from independent triplicate experiments.

The reducing power of constituents provides an imperative indicator of its prospective antioxidant potential. The FRAP assay is based on a transition from yellow to green associated with power of the sample to reduce a Fe^3+/^ferricyanide complex to Fe^2+^. Consequently, the percentage of Fe^2+^ reduced can be determined by measuring the absorbance at 700 nm [28]. The reducing powers were 6.78–23.50, 10.80–17.67, and 6.46–12.97 Fe^2+^g^−1^ for the essential oil and ethanolic and methanolic extracts, respectively, from wild accessions of *O. vulgare* L. For cultivated accessions of *O. vulgare,* the reducing power was 10.30–14.50, 5.60–10.60, and 1.90–4.80 Fe^2+^g^−1^ for the essential oil and ethanolic and methanolic extracts, respectively, as shown in Table 9. Free radical-scavenging potential of reference standards like arbutin, carvacrol, terpinen-4-ol, *β*-caryophyllene, ocimene, sabinene, and thymol standard compounds or pure compound was evaluated and represented in Figure 1.

**Figure 2:**
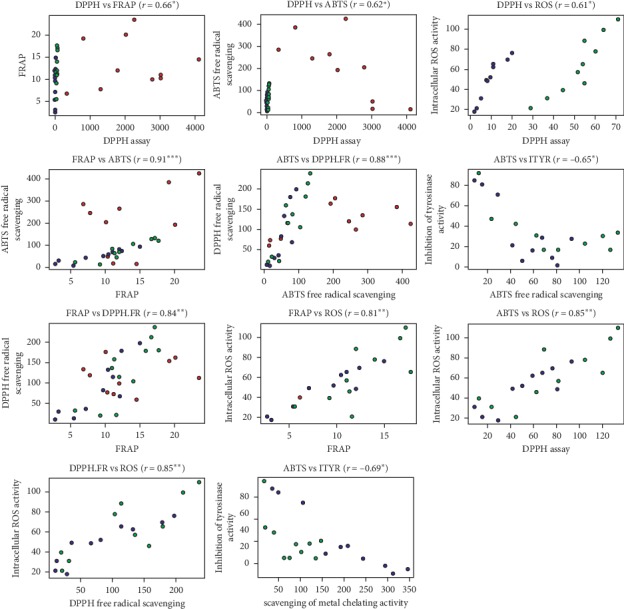
Representation of correlation between different assays including cellular tyrosinase, ABTS, DPPH, and FRAP free radical-scavenging, and intracellular ROS activities (%).

In this study, we evaluated the effect of carvacrol, thymol, terpinen-4-ol, caryophyllene, ocimene, sabinene on cell viability, ABTS^+^ levels, DPPH levels, FRAP free radical-scavenging, and intracellular ROS activities (%). The variation in biological efficacy of the samples agreed with the variation in percentage of reference compounds in the samples. Correlation between different assays to determine free radical-scavenging activity has been represented in Figure 2.

**Figure 3:**
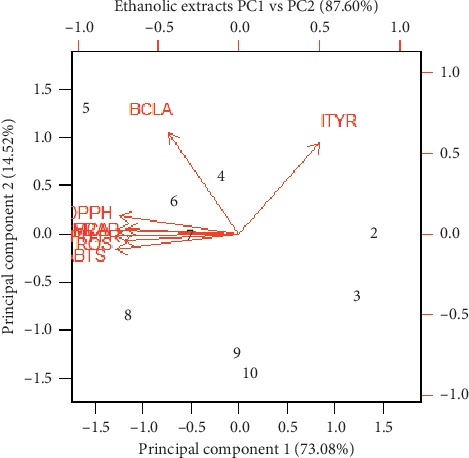
Representation of correlation between different assays including cellular tyrosinase, ABTS, DPPH, and FRAP free radical-scavenging, and intracellular ROS activities (%) by ethanolic extracts of *Origanum vulgare* L.

**Figure 4:**
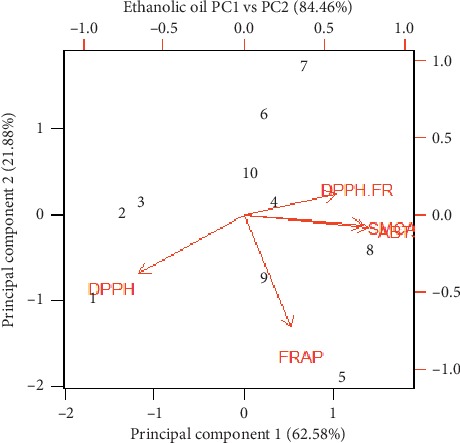
Representation of correlation between different assays including cellular tyrosinase, ABTS, DPPH, and FRAP free radical-scavenging, and intracellular ROS activities (%) by essential oil of *Origanum vulgare* L.

**Figure 5:**
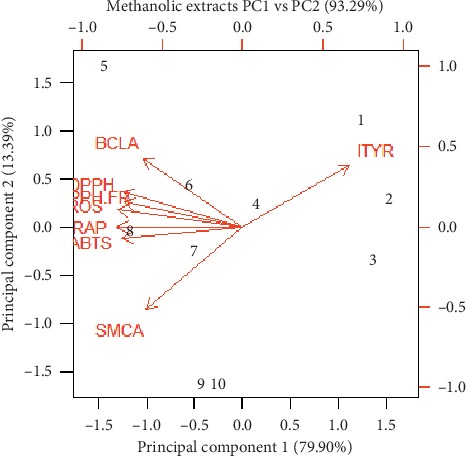
Representation of correlation between different assays including cellular tyrosinase, ABTS, DPPH, and FRAP free radical-scavenging, and intracellular ROS activities (%) by methanolic extracts of *Origanum vulgare* L.

Three principal coordinate analyses (PCAs) were used to describe the relationship between different wild and cultivated accessions of *O. vulgare* L., as shown in Figure 3. Graphs of the PCA biplot in Figures 3–5 reveal the comparative efficacy of different assays as determined by the eigenvalue. Methanolic extracts exhibited the greatest correlation (93.29%) between principal component 1 (DPPH) and principal component 2 (FRAP) followed by ethanolic extracts 87.60% and essential oils (82.46%). The greatest eigenvalue was found in the DPPH assay of the methanolic extracts (6.39) followed by ethanolic extracts 5.847 and 3.12 in essential oil. The lowest eigenvalue (0.006) was found for component 7 inhibition in tyrosinase activity in methanolic extracts.

### 3.2. Metal Scavenging Capacity of Wild and Cultivated Accessions of *Origanum vulgare L*

The chelating potential of ferrous ions by wild and cultivated accessions of *O. vulgare* was determined. Cultivated and wild accessions of *O. vulgare* L. exhibited considerably a lower metal scavenging activity than the positive control EDTA. Metal chelating activities were 50.20–119.8% and 62.9–140.9% in methanolic and ethanolic extracts from wild accessions of *O. vulgare* L., respectively. For cultivated accessions, the metal chelating activities were 11–35% and 23–90% in methanolic and ethanolic extracts, respectively, as shown in Table 10. The positive control EDTA exhibited an appreciably higher value of 345.5%.

#### 3.2.1. Cell Culture and Intracellular ROS Activity (%) of Wild and Cultivated Accessions of *O. vulgare* L

The theory underlying this assay is that DCFH-DA disseminates via the cell membrane and subsequently undergoes enzymatic esterification to release DCFH, which reacts with ROS to produce DCF, a fluorescent product. When categorized DCFH-DA was converted from DCFH-DA to DCFH and confined within the cell when incubated with H_2_O_2_, the fast intensity amplification of DCF fluorescence signified the oxidation of DCFH by intracellular radicals [30]. An extensive range of H_2_O_2_ was employed (0.002, 0.006, 0.02, 0.03, 0.3, 0.7, and 1.5 mM) to provoke oxidative stress to cell line, as shown in Table 9. Following 1 h of incubation with 0–0.7 mM H_2_O_2_ (at a concentration of 2 × 10^4^ cells/ml), no significant change was observed, but at a concentration of 1.5 mM, H_2_O_2_ diminished cell viability by 18.45%. Comparative levels of ROS displayed a significant decline with the highest value at 4 *μ*g/ml wild *O. vulgare* and 10 *μ*g/ml cultivated *O. vulgare* suppressing intracellular ROS production in C6 cells with approximately 89.8% and 102.34% of H_2_O_2_-treated cells, respectively. Moreover, the percent inhibition of cellular ROS activities in C6 cell lines by *O. vulgare* surpassed those caused by L-ascorbic acid or trolox. A similar assay was carried out with the reference compounds carvacrol, thymol, terpinen-4-ol, caryophyllene, ocimene, and sabinene. The viability ability of each compound followed the order carvacrol > sabinene > caryophyllene > terpinen-4-ol > ocimene > thymol, as shown in Figure 1.

#### 3.2.2. Inhibition of Tyrosinase Activity and Linoleic Acid Oxidation Properties of Wild and Cultivated Accessions of *O. vulgare* L

Numerous compounds have been screened to establish their efficacy in suppressing melanogenesis, including the tyrosinase inhibitors arbutin, hydroquinone, and kojic acid. However, hydroquinone can cause skin toxicity and kojic acid can cause tumors [31]. Therefore, research has focused on substitutes to treat hyperpigmentation, and safe dosage *in vitro* must always be evaluated prior to skin whitening assays. This study compared the skin whitening effect of various concentrations of wild and cultivated *O. vulgare* relative to the standard agents arbutin and *L*-ascorbic acid in human skin fibroblast Hs68 cells using a tyrosinase inhibition assay. Results from treatment with 10–15 *μ*g/ml arbutin are shown in Figure 1. Samples from wild and cultivated accessions of *O. vulgare* L. were applied to Hs68 cells at concentrations of 10–20 *μ*g/ml, and tyrosinase inhibition was observed. As shown in Table 6, a greater antityrosinase activity was observed in methanolic extracts of cultivated *O. vulgare* L. (48–81.1%) than in methanolic extracts of wild accessions (6–31%). Similar observations were recorded with ethanolic extracts in wild and cultivated accessions.

In the linoleic acid inhibition assay, both the methanolic and ethanolic extracts were effective in inhibiting linoleic acid oxidation. Among wild accessions of *O. vulgare*, the ethanolic extract (30 *μ*g/ml) caused 10.78–46.90% inhibition, while in cultivated accessions, it caused 10.12–26.72% inhibition. Methanolic extracts of wild accessions caused 18.30–46.72% inhibition, while the equivalent extracts from cultivated accessions caused 8.37–22.77% inhibition, as shown in Table 6.

#### 3.2.3. Antimicrobial Potential of Wild and Cultivated Accessions of *O. vulgare* L

The antimicrobial abilities of essential oil and methanolic extracts procured from wild and cultivated accessions of *O. vulgare* against diverse microbes were evaluated, and the efficacies were qualitatively and quantitatively evaluated by the incidence or lack of inhibition zones, zone diameter, and MIC values. The observations are shown in Tables 2 and 3. The observations demonstrate the significant efficacy of essential oil from both cultivated and wild accessions of *O. vulgare* against the 10 bacteria, 15 fungi, and yeast species analyzed. However, the cultivated accessions exhibited comparatively lower activity. Bacteria were the ones most affected by *O. vulgare,* with maximal inhibition zones and MIC values in the range of 12–35 mm and 13.78–128 *μ*l/ml (Tables 2 and 3) after treatment with wild accessions of *O. vulgare* L. The maximal inhibition zones and MIC values of the yeast and fungi species on the essential oil of *O. vulgare* were 14–38 mm and 30.15–137 *μ*l/ml, respectively (Tables 2 and 3). In case of cultivated accessions of *O. vulgare*, the maximal inhibition zones and MIC values for bacterial strains procured from wild accessions of *O. vulgare* were in the range of 14–27 mm and 17.19–147 *μ*l/ml (Tables 4 and 5), respectively. Maximal inhibition zones and MIC values of essential oil on yeast and fungi species were 14–38 mm and 30.15–137 *μ*l/ml, respectively (Tables 4 and 5).

## 4. Discussion

### 4.1. Chemical Composition of the Essential Oil

Previous research has reported that the genus *Origanum* exhibits two chemotypes with different concentrations of monoterpenes, such as terpinen-4-ol, *cis*- and *trans*-sabinene hydrate, carvacrol, and thymol [32–36]. The variability in essential oil composition of *O. vulgare* L. is due to diverse subspecies as well as different ecological and climatic parameters [6, 37, 38]. We found a variation in essential oil content even among cultivated accessions ranging from 52.99% to 62.05%. Moreover, various compounds in the volatile fraction were not identified in contrast to wild accessions, which had a broader spectrum of constituents. Some chemical constituents, like *p*-cymene, were absent in cultivated accessions but present in wild accessions in meagre concentrations. *Origanum vulgare* L. is a highly variable species having different chemotypes and exploitation of genetic variability that can be utilized for development of new cultivars rich in monoterpene and sesquiterpene [32, 33]. This disparity in essential oil components in genotypes within different habitats can be correlated with different gene expressions of key enzymes implicated in terpene biosynthesis [39, 40]. The disparity in essential composition can be due to diverse geographical location [1], varied climatic zones [41], and different ecological variables [42]. Variation in essential oil components is also dependent on origin of plants [43], stage of harvest [44], and intrinsic factors, for example, sexual polymorphism or genetic mechanism specifically phenolic compounds [45]. Though genetic and chemodiversity of essential oil profiles is extensively explored throughout world [22, 46], more efforts are required for deciphering the variation among genotypes of genus *Origanum* in Indian Himalayas so as to improve exploitation of wild local population of oregano for commercialization [47].

### 4.2. Free Radical-Scavenging Capacity and Reducing Power of Wild and Cultivated Accessions of *O. vulgare* L

Assessment of the antioxidant properties of cultivated and wild accessions of *O. vulgare* was performed using several in vitro assays, which are based on free radical scavenging. In this study, we chose DPPH, FRAP, and ABTS^+^ radicals as prime substrates to analyze the antioxidant properties of essential oil and plant extracts owing to the stability of these radicals. Trolox equivalent antioxidant capacity TEAC is an estimate of scavenging ABTS^+^ radical cation ability and its comparison with reference compound trolox, as described in Table 9. Antioxidant potential of plant extract is estimated by its capacity to quench free radicals using corresponding FRAP and ABTS^+^ radical-scavenging potentials. The antioxidant potential of *Origanum* extract can be correlated with surplus of phenolic compounds. Numerous studies have demonstrated linear correlation between monoterpenes fractions and antioxidant activities of *Origanum* extracts [5, 48, 49]. Methanolic extracts exhibited a highest antioxidant activity due to a higher dielectric constant/polarity and hence categorized as ideal solvent for extraction. Our studies demonstrate linear correlation between DPPH, FRAP, and ABTS with antioxidant potential of *Origanum* extracts and essential oil [50–52]. A higher proportion of volatile terpene fraction validates antioxidant potential as we have evaluated each monoterpene was subjected to the same assay to determine their competence in antioxidant potential.

### 4.3. Metal Scavenging Capacity of Wild and Cultivated Accessions of *Origanum vulgare* L

Complexation with Fe^2+^ can form ferrozine, which exhibits chelating activity and inhibits metal complexation. Consequently, the amount of color change is directly proportional to the chelating activity of the samples being tested. Methanolic and ethanolic extracts of genus *Origanum* exhibit significantly high metal chelating activity, which may be due to reduction of metals by antioxidant compounds like linalool [53, 54]. Metal chelating capacity of phytochemicals is due to high level of hydroxyl substitution by monoterpenes [55].

### 4.4. Cell Culture and Intracellular ROS Activity (%) of Wild and Cultivated Accessions of *O. vulgare* L

The free radical-scavenging potentials of wild and cultivated accessions of *O. vulgare* were investigated by studying the sample capacities to quench free radicals in cell cultures. Levels of intracellular ROS were measured by fluorescence of DCF in a DCFH-DA assay. The direct free radical-scavenging potential by *Origanum vulgare* can be demonstrated by significantly stronger antioxidant activity exhibited by phenolic groups in *Origanum* extracts, which inhibit chain initiation by declining generation of lipid peroxyl free radicals along with scavenging of superoxide and hydroxyl radicals [56, 57].

### 4.5. Inhibition of Tyrosinase Activity and Linoleic Acid Oxidation Properties of Wild and Cultivated Accessions of *O. vulgare* L

Majority of antioxidants are known to inhibit melanogenesis by scavenging ROS and by declining o-quinones implicated in biosynthesis of melanin biosynthesis thereby impeding oxidative polymerization. Current investigation describes antioxidant potential of *Origanum* extracts through free radical scavenging which may be correlated to the inhibition of melanogenesis [5, 58]. Melanogenesis is the multistep process involving enzymatic oxidation of L-DOPA to its corresponding o-dopaquinone, catalysed by tyrosinase. Hence, any impediment in tyrosinase activity can inhibit melanogenesis. Present work describes potential of inhibition of tyrosinase activity by *Origanum* extracts, and the percentage inhibition of cellular tyrosinase activity was significantly higher in cultivated accessions, which may be due to higher levels of arbutin [59, 60]. Arbutin is an established inhibitor of tyrosinase activity and its higher concentration in cultivated accessions to prove its potential in inhibiting melanogenesis [61].

In the case of inhibition of linoleic acid assay, both methanol and ethanolic extracts exhibited only the linoleic acid oxidation 46.72% and 46.92% inhibitions at 2 mg/ml concentrations, respectively, that were significantly lower than positive control BHT. However, this activity can be significantly improved by evaluating higher concentrations of the same extract. The greatest activity caused by the essential oil can be attributed to the presence of more active constituents in the volatile fractions including a higher percentage of carvacrol, thymol, and *β*-caryophyllene. Supporting this theory are numerous research reports that confirm the significant role of carvacrol, thymol, and *β*-caryophyllene in antioxidant defense systems [62–65].

### 4.6. Antimicrobial Potential of Wild and Cultivated Accessions of *O. vulgare* L

Though we have evaluated methanolic extracts of both wild and cultivated accessions, there was no or very low activity as observed from decreased rate of decline in inhibition zones and MIC values. Following above observations, it can be concluded that the essential oil has a higher antimicrobial potential owing to elevated levels of carvacrol and thymol, as determined by GC analysis. Carvacrol and thymol are significant phenolic monoterpenoids that exhibit numerous pharmacological activities such as antimicrobial, antitumor, and insecticidal properties [66, 67]. The present study describes antimicrobial activity against food pathogens including *Enterobacter* spp., *Bacillus* spp., *Salmonella* spp., *Staphylococcus aureus*, *Candida* spp., *Fusarium* spp., *Aspergillus* spp., *Rhizopus* spp., and *Penicillium* spp. isolates. Perusal of the data found in Table 1 ascertains the presence of numerous other compounds exhibiting antipathogenic activities, such as caryophyllene and germacrene-D. Thus, the results may signify that *O. vulgare* possesses more compounds with antimicrobial and antioxidant potential, which can be utilized for food preservation and/or extension of shelf-life. In addition, the data in the present study support the use of *O. vulgare* plants for tea preparation or as food additives and in traditional remedies for the treatment of infectious diseases.

## 5. Conclusion

This study concludes that the methanolic extracts of *O. vulgare* are more effective as an antioxidant agent than the ethanolic extracts even at the concentration of the 3 *μ*g/ml. However, essential oil from *O. vulgare* L. is more potent as an antimicrobial agent due to the higher concentration of the carvacrol and thymol. Methanolic extracts of *O. vulgare* exhibited high depigmentation potential about from 81.0% to 87.0%, while ethanolic extracts display higher inhibition of reactive oxygen species ranging from 88.54 to 99.02%. Therefore, the study resolves the efficacy of essential oil against microbial pathogenesis, methanolic extracts as potent depigmentation agents, and ethanolic extracts as potent free radical scavenger.

## Figures and Tables

**Table 1:** Collection and procurement of material from different naturalized and cultivated populations across north western Himalayas.

Place		North	Altitude	Total Ascent	PAR	UV	Temperature
		East	ft		(*μ*mol photons m^−2^·s^−1^)	(*μ*mol m^−2^·s^−1^)	August-September
*Cultivated populations of Oreganum vulgare L.*	Tangmarg (Yarikhah)	34°03	8400	2650	12431	78	Max-18
		74°25					Min-11
	Pulwama	34°05	5400	1196	1045	83	Max-24
	(Bonera)	74°38					Min-13
	Sanatnagar	34°90	5426	4,967	1123	112	Max-28
		74°54					Min-14

*Wild populations of Oreganum vulgare L.*	Churwan	34°38	8027	15345	16821	113.1	Max-15
	(Gurez)	74.51					Min-8
	Kanzalwan	34°38	8000	5277	1600	117	Max-17
	(Gurez)	74°43					Min-10
	Markoot	34°.38	7945	15341	1496	107	Max-18
	(Gurez)	74°57					Min-9
	Izmarg	34°31	7875	15420	1015.7	109.7	Max-20
	(Gurez)	74°41					Min-12
	Dachigam	34°81	9500	1296	1012	104	Max-12
		75°20					Min-6
	Uri	33.83	7861	1431	1298	95	Max-20
		75.30					Min-13
	Yusmarg	34.08	4472	1023	948	73	Max-17
		74.03					Min-9

**Table 2:** Antimicrobial activity of wild accessions of *Origanum vulgare L.* extract and essential oil against the bacterial strains tested based on the disc diffusion method and MIC value.

Antimicrobial activity	EO (10 *μ*l/disc)	Standard antibiotic discs	EO	Standard drug (maxipime)
*Acinetobacter baumannii-*A8	18	18 mm (OFX)	13.78	31.25
*Bacillus macerans-*M58	28	19 mm (OFX)	31.25	15.62
*Bacillus megaterium-*M3		9 mm (SCF)	128	15.62
*Bacillus subtilis-*ATCC-6633	29	28 mm (SCF)	31.25	62.5
*Bacillus subtilis-*A57		28 mm (OFX)	62.5	125
*Brucella abortus-*A77		12 mm (OFX)		15.62
*Burkholderia cepacia-*A225		22 mm (SCF)		31.25
*Clavibacter michiganensis-*A227	35	25 mm (SCF)	15.62	31.25
*Enterobacter cloacae-*A135		20 mm (NET)		62.5
*Enterococcus faecalis-*ATCC-29122	10	18 mm (SCF)	62.5	125
*Escherichia coli-*A1	18	10 mm (SCF)	31.25	125
*Klebsiella pneumoniae-*A137		12 mm (OFX)		31.25
*Proteus vulgaris-*A161		12 mm (OFX)		15.62
*Proteus vulgaris-Kukem-*1329	12	13 mm (OFX)	62.5	125
*Pseudomonas aeruginosa-*ATCC-9027		13 mm (NET)		62.5
*Pseudomonas aeruginosa-*ATCC-27859		22 mm (NET)		31.25
*Pseudomonas syringae pv. tomato-*A35		22 mm (OFX)		62.5
*Salmonella enteritidis-*ATCC-13076		24 mm (SCF)		15.62
*Staphylococcus aureus-*A215	14	27 mm (SCF)	62.5	
*Staphylococcus aureus-*ATCC-29213		22 mm (SCF)		31.25
*Staphylococcus epidermis-*A233		22 mm (SCF)		62.25
*Streptococcus pyogenes-*ATCC-176	12	10 mm (OFX)	62.5	
*Streptococcus pyogenes-Kukem-*676	19	13 mm (OFX)	31.25	
*Xanthomonas campestris-*A235		20 mm (SCF)		31.25

OFX = ofloxacin (10 *μ*g/disc), SCF = sulbactam (30 *μ*g) + cefoperazona (75 *μ*g) (105 *μ*g/disc), and NET = netilmicin (30 *μ*g/disc) were used as positive reference standard antibiotic disc.

**Table 3:** Antimicrobial activity of wild accession *Origanum vulgare L*. extract and essential oil against the yeast and fungi isolates tested based on the disc diffusion method and MIC value.

Yeast and fungi species	EO	MIC values	Standard drug (amphotericin B)
Candida albicans-A117	17 mm	127	31.25
*Fungi*			
Alternaria alternata	38 mm	135	15.62
Aspergillus flavus	24 mm	135	15.62
Aspergillus variecolor	32 mm	32.75	62.5
Fusarium acuminatum	27 mm	62.5	62.5
Fusarium oxysporum	24 mm	62.5	62.5
Fusarium solani	18 mm	31.25	62.5
Fusarium tabacinum	35 mm	62.5	15.62
Monilinia fructicola	14 mm	15.62	31.25
Penicillium spp.	35 mm	31.25	125
Rhizopus spp.	14 mm	125	31.25
Rhizoctonia solani	35 mm	33.25	62.5
Scloretinia sclerotiorum	16 mm	32.25	125
Scloretinia minor	27 mm	30.15	31.25
Trichophyton mentagrophytes	35 mm	32.25	15.62
Trichophyton rubrum	29 mm	30.25	15.62

Inhibition zone in diameter (mm) around the discs impregnated with 10 *μ*l of essential oil and extracts (300 *μ*g/disc).

**Table 4:** Antimicrobial activity of cultivated accessions of *Origanum vulgare L.* extract and essential oil against the bacterial strains tested based on the disc diffusion method and MIC value.

Antimicrobial activity	EO (10 *μ*l/disc)	Standard antibiotic discs	EO	Standard drug (maxipime)
*Acinetobacter baumannii-A8*	18	18 mm (OFX)	13.78	31.25
*Bacillus macerans-*M58	28	19 mm (OFX)	31.25	15.62
*Bacillus megaterium-*M3		9 mm (SCF)	128	15.62
*Bacillus subtilis-*ATCC-6633	29	28 mm (SCF)	31.25	62.5
*Bacillus subtilis-*A57		28 mm (OFX)	62.5	125
*Brucella abortus-*A77		12 mm (OFX)		15.62
*Burkholderia cepacia-*A225		22 mm (SCF)		31.25
*Clavibacter michiganense-*A227	35	25 mm (SCF)	15.62	31.25
*Enterobacter cloacae-*A135		20 mm (NET)		62.5
*Enterococcus faecalis-*ATCC-29122	10	18 mm (SCF)	62.5	125
*Escherichia coli-*A1	18	10 mm (SCF)	31.25	125
*Klebsiella pneumoniae-*A137		12 mm (OFX)		31.25
*Proteus vulgaris-*A161		12 mm (OFX)		15.62
*Proteus vulgaris-Kukem-*1329	12	13 mm (OFX)	62.5	125
*Pseudomonas aeruginosa-*ATCC-9027		13 mm (NET)		62.5
*Pseudomonas aeruginosa-*ATCC-27859		22 mm (NET)		31.25
*Pseudomonas syringae pv. tomato-*A35		22 mm (OFX)		62.5
*Salmonella enteritidis-*ATCC-13076		24 mm (SCF)		15.62
*Staphylococcus aureus-*A215	14	27 mm (SCF)	62.5	
*Staphylococcus aureus-*ATCC-29213		22 mm (SCF)		31.25
*Staphylococcus epidermis-*A233		22 mm (SCF)		62.25
*Streptococcus pyogenes-*ATCC-176	12	10 mm (OFX)	62.5	
*Streptococcus pyogenes-Kukem-*676	19	13 mm (OFX)	31.25	
*Xanthomonas campestris-*A235		20 mm (SCF)		31.25

OFX = ofloxacin (10 *μ*g/disc), SCF = sulbactam (30 *μ*g) + cefoperazona (75 *μ*g) (105 *μ*g/disc), and NET = netilmicin (30 *μ*g/disc) were used as positive reference standard antibiotic disc.

**Table 5:** Antimicrobial activity of cultivated accessions of *Origanum vulgare L*. extract and essential oil against the yeast and fungi isolates tested based on the disc diffusion method and MIC value.

Yeast and fungi species	EO	MIC values	Standard drug (amphotericin B)
Candida albicans-A117	17	127	31.25
*Fungi*			
Alternaria alternata	20	135	15.62
Aspergillus flavus	24	135	15.62
Aspergillus variecolor	24	32.75	62.5
Fusarium acuminatum	27	62.5	62.5
Fusarium oxysporum	24	62.5	62.5
Fusarium solani	18	31.25	62.5
Fusarium tabacinum	26	62.5	15.62
Monilinia fructicola	14	15.62	31.25
Penicillium spp.	35	31.25	125
Rhizopus spp.	12	125	31.25
Rhizoctonia solani	25	33.25	62.5
Scloretinia sclerotiorum	16	32.25	125
Scloretinia minor	24	30.15	31.25
Trichophyton mentagrophytes	25	32.25	15.62
Trichophyton rubrum	29	30.25	15.62

Inhibition zone in diameter (mm) around the discs impregnated with 10 *μ*l of essential oil and extracts (300 *μ*g/disc).

**Table 6:** Comparative biological efficacy of plant extracts of cultivated and wild accessions of *Origanum vulgare* L., thriving across north western Himalayas.

Inhibition of tyrosinase activity (%)			*β*-Carotene-linoleic acid assay	
Places	METH	ETH	METH	ETH
Tangmarg	81.0 ± 1.75	87.0 ± 1.93	22.77 ± 0.47	26.72 ± 0.55
Bonera	71.0 ± 1.66	47.0 ± 0.88	10.52 ± 0.22	20.23 ± 0.42
Sanatnagar	48.0 ± 1.06	37.8 ± 0.68	8.37 ± 0.17	10.12 ± 0.20
Chorwan	31.0 ± 0.56	31.0 ± 0.62	33.04 ± 0.74	36.73 ± 0.80
Kanzalwan	27.0 ± 0.42	21.0 ± 0.47	46.72 ± 0.99	46.90 ± 1.11
Markoot	30.4 ± 0.67	29.4 ± 0.58	35.23 ± 0.73	37.08 ± 0.77
Izmarg	16.6 ± 0.36	23.1 ± 0.47	27.12 ± 0.56	32.26 ± 0.67
Dachigam	9.2 ± 0.18	17.1 ± 0.42	30.69 ± 0.66	17.09 ± 0.40
Yusmarg	1.9 ± 0.03	17.0 ± 0.36	20.68 ± 0.43	10.78 ± 0.22
Uri	6.0 ± 0.12	16.9 ± 0.32	18.30 ± 0.38	16.98 ± 0.14
CD	16.22	15.73	0.76	0.92
SE (d)	7.66	7.43	0.36	0.43
SE (M)	5.41	5.25	0.25	0.31
CV	21.90	22.18	1.70	2.10

**Table 7:** Essential oil composition in diverse naturalized and cultivated oregano populations.

Compounds	Tangmarg (Yarikhah)	Pulwama (Bonera)	Sanatnagar	Chorwan (Gurez)	Kanzalwan (Gurez)	Markoot (Gurez)	Izmarg (Gurez)	Dachigam	Uri	Yusmarg
*α*-Thujene	0.01	0.02	—	0.04	0.03	0.03	0.02	0.02	0.03	0.01
*α*-Pinene	0.01	0.02	—	0.04	0.03	0.03	0.02	0.02	0.04	0.01
Camphene	0.01	0.02	—	0.04	0.03	0.03	0.02	0.02	0.03	0.01
Sabinene	0.07	0.04	0.01	0.02	0.08	0.02	0.01	0.06	0.08	0.04
*β*-Pinene	0.01	0.01	—	0.02	0.03	0.01	0.02	0.02	0.03	0.01
1-Octen-3-ol	—	—	—	0.01	0.03	0.03	0.01	0.03	0.03	0.01
3-Octanone	—	—	—	0.02	0.01	0.02	0.03	0.01	0.01	0.009
Myrcene	0.02	0.02	0.01	0.07	0.12	0.13	0.13	0.09	0.09	0.06
*α*-Phellandrene	—	—	—	0.03	0.09	0.08	0.03	0.07	0.03	0.05
3-Carene	—	—	—	0.01	0.14	0.01	0.12	0.09	0.08	0.04
*α*-Terpinene	—	—	—	0.08	0.04	0.02	0.02	0.09	0.04	0.02
*p*-Cymene	—	—	—	0.03	1.23	0.08	0.04	0.09	0.09	0.07
Limonene	0.02	0.04	0.07	0.14	0.19	0.09	0.10	0.03	0.04	0.09
*β*-Phellandrene	—	—	—	0.02	0.06	0.03	0.09	0.02	0.03	0.01
(Z)-*β* Ocimene	—	—	—	0.12	0.18	0.14	0.12	—	—	—
(E)-*β* Ocimene	—	—	—	0.10	0.08	0.12	0.14	—	—	—
*β*-Terpinene	—	—	—	—	0.09	—	—	0.03	0.02	0.07
*cis*-Sabinene hydrate	0.01	0.02	0.04	0.9	1.02	1.03	1.05	0.73	0.69	0.58
Terpin-olen	—	—	—	0.09	0.04	0.02	0.03	0.02	0.04	0.09
*trans*-Sabinene hydrate	0.01	0.02	0.04	0.9	1.43	1.01	0.98	1.02	0.08	1.0
Borneol	—	—	—	0.01	0.03	0.03	0.01	0.03	0.09	0.04
Terpinene-4-ol	—	0.03	0.02	0.32	0.24	0.19	0.22	0.17	0.13	0.12
*α*-Terpineol	—	—	—	0.02	0.03	0.01	0.02	0.02	0.05	0.02
*trans*-Dihydrocarvone	0.01	0.02	—	0.04	0.03	0.03	0.02	0.02	0.01	0.04
Carvacrol methylether	0.07	0.04	0.01	0.02	0.08	0.02	0.01	0.06	0.04	0.08
Thymoquinone	0.02	0.04	0.07	0.14	0.19	0.09	0.10	0.034	0.05	0.03
Thymol	0.08	0.03	0.04	0.01	0.04	0.03	0.13	3.3	2.5	1.08
Carvacrol	52.3	61.09	58.78	76.5	84.54	79.89	80.12	82.72	78.09	69.98
*β*-Caryophyllene	0.01	0.04	0.03	0.14	1.23	1.45	1.87	0.69	0.53	0.42
*α*-Humulene	0.04	00.3	0.02	0.08	0.07	0.09	0.03	0.09	0.04	0.01
Allo-aromadendrene	0.01	0.02	—	0.04	0.03	0.03	0.02	0.02	0.03	0.01
*α*-Muurolol	0.01	0.02	—	0.04	0.03	0.03	0.02	0.02	0.04	0.02
*β*-Bisabolene	0.07	0.04	0.01	0.02	0.08	0.02	0.01	0.06	0.04	0.01
*γ*-Cadinene	0.02	0.04	0.07	0.14	0.19	0.09	0.10	0.034	0.013	0.07
*δ*-Cadinene	0.01	0.02	—	0.04	0.03	0.03	0.02	0.02	0.01	0.009
Spathulenol	0.07	0.04	0.01	0.02	0.08	0.02	0.01	0.06	0.04	0.02
Caryophyllene oxide	0.01	0.01	—	0.02	0.03	0.01	0.02	0.02	0.01	0.007
Epi-*α*-muurolol	0.07	0.04	0.01	0.02	0.08	0.02	0.01	0.06	0.03	0.01
*α*-Eudesmol	0.02	0.04	0.07	0.14	0.19	0.09	0.10	0.03	0.02	0.005
Total	**52.99**	**62.05**	**59.31**	**80.44**	**91.18**	**84.1**	**85.82**	**89.918**	**83.243**	**74.155**

**Table 8:** Comparative antioxidant potential of the essential oils and plant extracts of cultivated and wild accessions of *Origanum vulgare* L., thriving across north western Himalayas.

DPPH assay (IC50) *μ*g/ml				DPPH free radical scavenging (%)		
Places	EO	METH	ETH	EO	METH	ETH
Tangmarg	1300.2 ± 27.06	4.9 ± 0.08	44.5 ± 0.92	77 ± 1.60	28 ± 0.62	32 ± 0.68
Bonera	817.5 ± 17.05	2.9 ± 0.05	37.1 ± 0.77	73 ± 1.51	11 ± 0.88	18 ± 0.41
Sanatnagar	334.9 ± 6.95	0.9 ± 0.05	24.1 ± 0.62	60 ± 1.24	9 ± 0.12	15 ± 0.44
Chorwan	3021.0 ± 62.86	7.8 ± 0.15	55.0 ± 1.12	161.4 ± 3.63	169.6 ± 12.7	201 ± 4.42
Kanzalwan	4100.0 ± 85.34	19.9 ± 0.41	71.1 ± 1.45	175.4 ± 3.65	188 ± 28.72	222.6 ± 4.49
Markoot	2980.0 ± 62.65	10.9 ± 0.20	54.1 ± 1.12	157.4 ± 3.21	132.8 ± 12.76	179.4 ± 3.74
Izmarg	2265.4 ± 47.13	11.8 ± 0.32	60.1 ± 1.24	133.4 ± 2.76	114.4 ± 2.37	103.8 ± 2.17
Dachigam	1782.8 ± 37.10	17.9 ± 0.361	64.1 ± 1.34	119.4 ± 2.49	82.00 ± 1.17	147 ± 2.82
Yusmarg	2780.0 ± 57.87	7.9 ± 0.15	54.9 ± 1.12	161.4 ± 3.36	67.00 ± 2.92	114.6 ± 2.37
Uri	2021.0 ± 42.05	9.5 ± 0.20	51.7 ± 1.09	91.4 ± 2.05	52.0 ± 1.76	136.2 ± 2.82
CD	551.819	5.12	8.28	7.29	57.43	42.27
SE (d)	262.689	2.43	3.94	3.47	27.34	19.96
SE (M)	185.5	1.72	2.78	2.45	19.33	14.11
CV	14.87	33.00	9.46	4.25	40.34	21.44

CD, CV, and SE represent critical difference, coefficient of variation, and standard error.

**Table 9:** Comparative antioxidant potential of ethanolic and methanolic plant extracts of cultivated and wild accessions of *Origanum vulgare* as revealed by ABTS and FRAP assay.

FRAP (Fe^2+^g^−1^ of sample)				ABTS free radical scavenging (%)		
Places	EO	ETH	METH	METH	ETH	EO
Tangmarg	14.50 ± 0.30	10.60 ± 0.19	4.80 ± 0.11	8 ± 0.16	37 ± 0.92	15 ± 0.31
Bonera	11.09 ± 0.22	8.60 ± 0.11	2.70 ± 0.05	15 ± 0.31	23 ± 0.47	18 ± 0.37
Sanatnagar	10.30 ± 0.21	5.60 ± 0.09	1.90 ± 0.06	25 ± 0.60	12 ± 0.25	49 ± 1.02
Chorwan	12.03 ± 0.25	10.80 ± 0.24	6.46 ± 0.14	37.25 ± 0.86	55.87 ± 1.29	265.50 ± 5.52
Kanzalwan	23.50 ± 0.48	11.85 ± 0.23	14.28 ± 0.31	89.02 ± 1.94	127.64 ± 2.64	424.20 ± 8.83
Markoot	7.80 ± 0.16	17.67 ± 0.36	11.02 ± 0.23	67.45 ± 1.40	120.00 ± 2.49	245.10 ± 5.10
Izmarg	6.78 ± 0.14	13.97 ± 0.29	10.37 ± 0.21	58.82 ± 1.23	104.70 ± 2.18	284.90 ± 5.93
Dachigam	19.20 ± 0.40	16.08 ± 0.34	12.97 ± 0.25	80.39 ± 1.67	120.59 ± 2.45	384.40 ± 8.00
Yusmarg	20.90 ± 0.41	11.98 ± 0.25	11.98 ± 0.0.27	64.87 ± 1.47	81.76 ± 1.70	192.40 ± 4.00
Uri	10.09 ± 0.20	10.98 ± 0.22	9.70 ± 0.20	50.19 ± 1.04	68.81 ± 1.43	205.30 ± 4.27
CD	7.22	2.86	2.84	18.76	23.5	78.67
SE (d)	3.44	1.36	1.35	8.93	11.19	37.45
SE (M)	2.45	0.96	0.96	6.31	7.9	26.48
CV	33.10	13.25	20.47	23.39	18.27	23.07

CD, CV, and SE represent critical difference, coefficient of variation, and standard error.

**Table 10:** Comparative metal scavenging and intracellular ROS activity of plant extracts of cultivated and wild accessions of *Origanum vulgare* L., thriving across north western Himalayas.

Scavenging effect of metal chelating activity (%)				Intracellular ROS activity (%)	
Places	METH	EDTA	ETH	METH	ETH
Tangmarg	11 ± 0.29	23 ± 0.77	15 ± 0.36	27 ± 0.64	35 ± 0.82
Bonera	20 ± 0.40	50 ± 1.04	20 ± 0.41	21 ± 0.43	31 ± 0.64
Sanatnagar	35 ± 0.80	90 ± 2.02	32 ± 0.83	18 ± 0.37	21 ± 0.43
Chorwan	96.6 ± 2.13	140.3 ± 3.27	114.9 ± 2.5	49.4 ± 1.02	45.9 ± 0.95
Kanzalwan	119.8 ± 2.61	174.5 ± 3.98	140.9 ± 3.06	76.5 ± 1.59	109.86 ± 2.28
Markoot	76.98 ± 1.60	208.7 ± 4.33	88.9 ± 1.87	60.09 ± 1.35	67.22 ± 1.35
Izmarg	85 ± 1.75	242.9 ± 5.05	101.9 ± 2.13	62.43 ± 1.29	77.88 ± 1.62
Dachigam	108.2 ± 2.02	277.1 ± 6.12	127.9 ± 2.79	69.78 ± 1.45	99.2 ± 2.06
Yusmarg	50.2 ± 1.04	311.3 ± 6.48	62.9 ± 1.30	51.89 ± 1.08	88.54 ± 1.84
Uri	61.8 ± 1.30	345.5 ± 7.19	75.9 ± 1.57	48.78 ± 1.01	56.78 ± 1.18
CD	20.07	20.88	23.51	9.96	25.85
SE (d)	9.48	9.86	11.10	4.70	12.08
SE (M)	6.70	6.97	7.85	3.32	8.54
CV	16.70	7.95	16.92	12.08	25.27

## Data Availability

The data used to support the findings of this study are available from the corresponding author upon request.
